# Supplementary material for: Genome-Wide Analysis and Expression Profiling of the JAZ Gene Family in Response to Abiotic Stress in Alfalfa
Source: Int J Mol Sci. 2025 May 14;26(10):4684. doi: 10.3390/ijms26104684 (PMC12111722; doi:10.3390/ijms26104684)
Supplement: Supplementary file 1 [file ijms-26-04684-s001.zip › Figure S.docx]

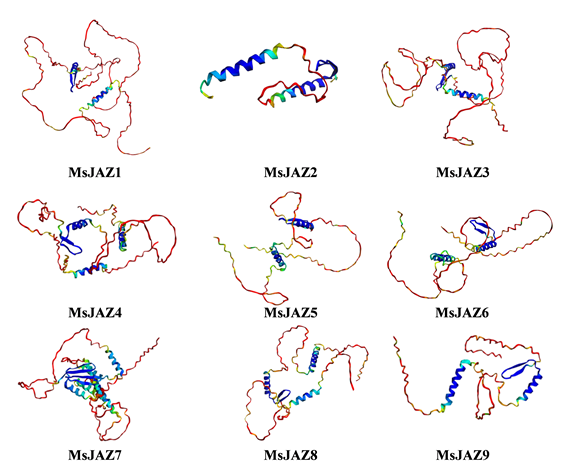


**Figure S1 3D structure and functional domains of MsJAZ proteins**


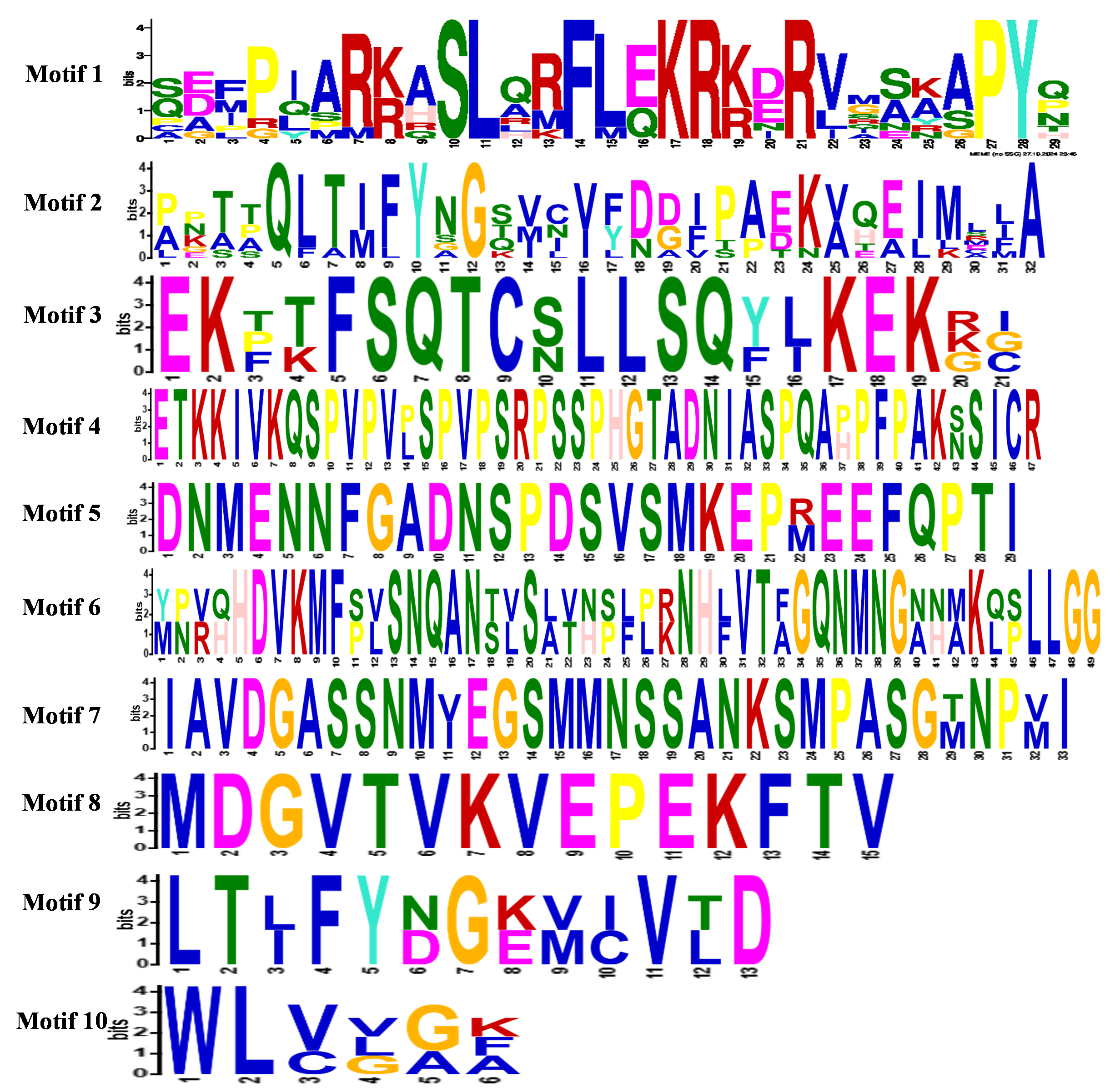


**Figure S2 The MEME motifs of MsJAZ proteins**

**
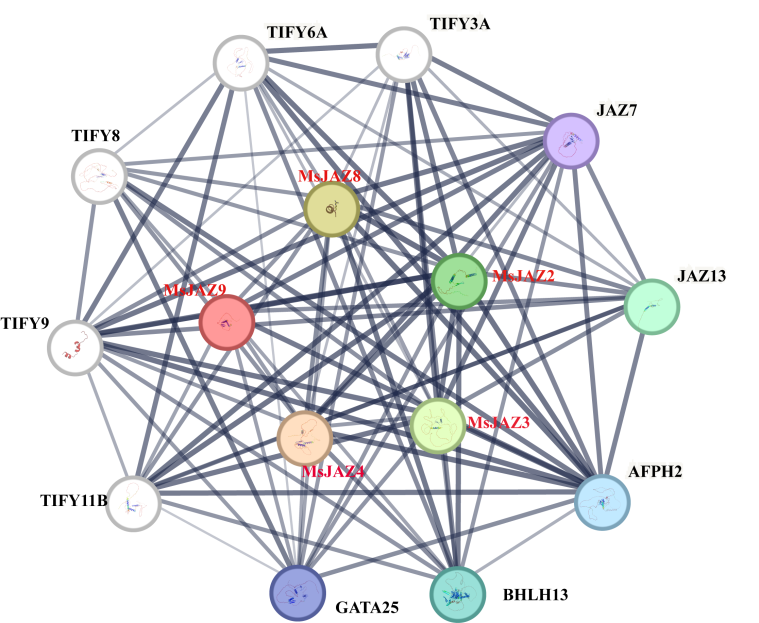
**


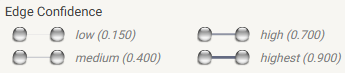


**Figure S3 MsJAZ Proteins Interaction Network**

**
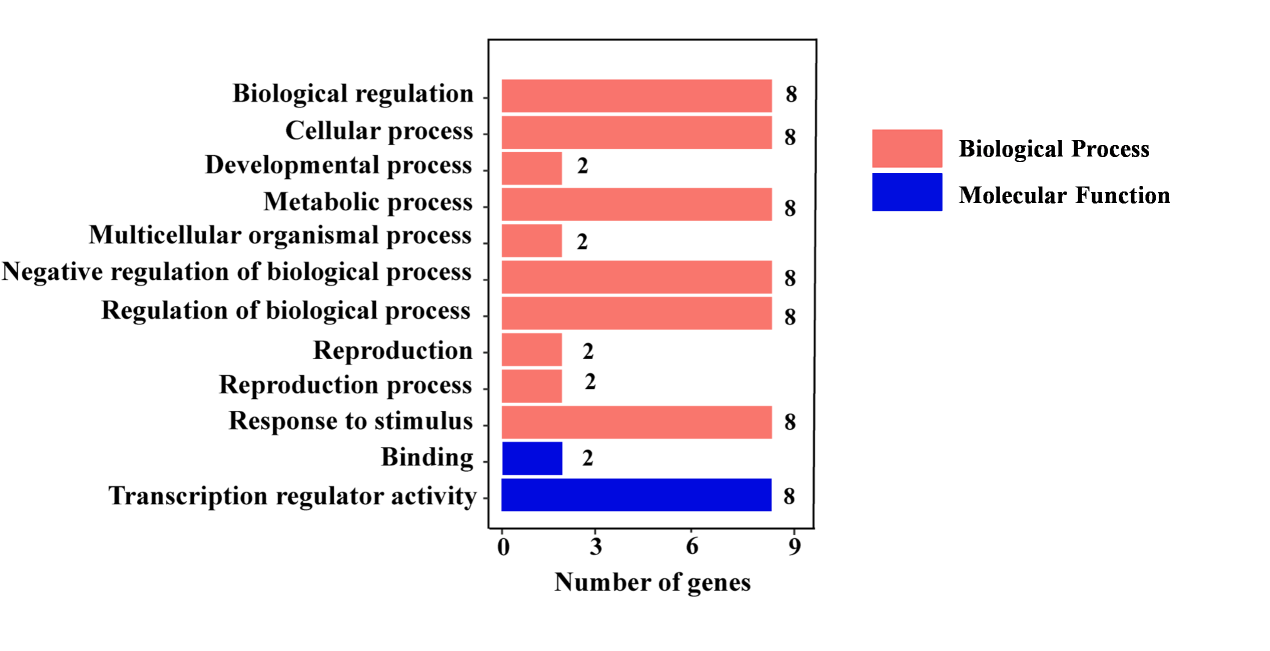
**

**Figure S4 Gene ontology (GO) annotation results of 9 *MsJAZ* genes**
